# Evaluating the Impact of a Point-of-Care Cardiometabolic Clinical Decision Support Tool on Clinical Efficiency Using Electronic Health Record Audit Log Data: Algorithm Development and Validation

**DOI:** 10.2196/38385

**Published:** 2022-09-06

**Authors:** Xiaowei Yan, Hannah Husby, Satish Mudiganti, Madina Gbotoe, Jake Delatorre-Reimer, Kevin Knobel, Andrew Hudnut, J B Jones

**Affiliations:** 1 Center for Health Systems Research Sutter Health Walnut Creek, CA United States; 2 Department of Clinical Informatics NorthBay Healthcare Fairfield, CA United States; 3 Sutter Gould Medical Foundation Sutter Health Modesto, CA United States; 4 Sutter Medical Group Sutter Health Sacramento, CA United States

**Keywords:** digital health, electronic health record, EHR audit logs, workflow efficiency, cardiometabolic conditions

## Abstract

**Background:**

Electronic health record (EHR) systems are becoming increasingly complicated, leading to concerns about rising physician burnout, particularly for primary care physicians (PCPs). Managing the most common cardiometabolic chronic conditions by PCPs during a limited clinical time with a patient is challenging.

**Objective:**

This study aimed to evaluate a Cardiometabolic Sutter Health Advanced Reengineered Encounter (CM-SHARE), a web-based application to visualize key EHR data, on the EHR use efficiency.

**Methods:**

We developed algorithms to identify key clinic workflow measures (eg, total encounter time, total physician time in the examination room, and physician EHR time in the examination room) using audit data, and we validated and calibrated the measures with time-motion data. We used a pre-post parallel design to identify propensity score–matched CM-SHARE users (cases), nonusers (controls), and nested-matched patients. Cardiometabolic encounters from matched case and control patients were used for the workflow evaluation. Outcome measures were compared between the cases and controls. We applied this approach separately to both the CM-SHARE pilot and spread phases.

**Results:**

Time-motion observation was conducted on 101 primary care encounters for 9 PCPs in 3 clinics. There was little difference (<0.8 minutes) between the audit data–derived workflow measures and the time-motion observation. Two key unobservable times from audit data, physician entry into and exiting the examination room, were imputed based on time-motion studies. CM-SHARE was launched with 6 pilot PCPs in April 2016. During the prestudy period (April 1, 2015, to April 1, 2016), 870 control patients with 2845 encounters were matched with 870 case patients and encounters, and 727 case patients with 852 encounters were matched with 727 control patients and 3754 encounters in the poststudy period (June 1, 2016, to June 30, 2017). Total encounter time was slightly shorter (mean −2.7, SD 1.4 minutes, 95% CI −4.7 to −0.9; mean –1.6, SD 1.1 minutes, 95% CI −3.2 to −0.1) for cases than controls for both periods. CM-SHARE saves physicians approximately 2 minutes EHR time in the examination room (mean −2.0, SD 1.3, 95% CI −3.4 to −0.9) compared with prestudy period and poststudy period controls (mean −1.9, SD 0.9, 95% CI −3.8 to −0.5). In the spread phase, 48 CM-SHARE spread PCPs were matched with 84 control PCPs and 1272 cases with 3412 control patients, having 1119 and 4240 encounters, respectively. A significant reduction in total encounter time for the CM-SHARE group was observed for short appointments (≤20 minutes; 5.3-minute reduction on average) only. Total physician EHR time was significantly reduced for both longer and shorter appointments (17%-33% reductions).

**Conclusions:**

Combining EHR audit log files and clinical information, our approach offers an innovative and scalable method and new measures that can be used to evaluate clinical EHR efficiency of digital tools used in clinical settings.

## Introduction

### Background

Approximately 34% of the population in the United States aged ≥18 years has a cardiometabolic condition (ie, diabetes mellitus [DM], hypertension, and high cholesterol), which are among the most common and costly health problems [1]. Managing these chronic conditions is an important area of focus for primary care physicians (PCPs) in the United States. However, the effective management of these chronic conditions can be challenging for patients and PCPs.

Patients spend <1% of their time with their PCPs and the rest on their own, attempting to adopt the care plan prescribed by their PCP into their daily lives [2]. During their limited time at the point of care with patients, PCPs rarely have enough time to review all the critical but scattered data in an electronic health record (EHR); given that, on average, PCPs only have 15 minutes with patients for a face-to-face visit [3]. Health care providers have expressed dissatisfaction with EHR systems [4-6] used to manage patients, which have generally been poorly designed for facilitating care delivery. Increasing evidence indicates that an EHR imposes an additional burden on physicians [7-10]. In particular, PCPs reported having the highest burnout associated with EHR use [11]. The causes of physician burnout are multifactorial, including the increasing complexity and cognitive burden of using the EHR and decreased face-to-face time with patients [12,13]. Moreover, longer EHR use time is negatively associated with patient satisfaction [14], especially with increased daytime EHR use, potentially occurring in the examination room, implying that physicians have less time to communicate with patients, which may adversely affect patient-physician relationships [15-17]. Therefore, technology or interventions that aim to reduce EHR time in the examination room can potentially improve health delivery quality from both physician and patient perspectives. Digital health solutions, including clinical decision support tools, hold the promise of helping physicians improve patient clinical outcomes or quality of care [18-21]; however, it is less clear whether these solutions have an impact on clinical EHR efficiency [3].

A well-designed and integrated digital tool can be sufficiently seamless so that the user feels unencumbered by the effort to open the additional platform and perceive the EHR and digital tool as one system [21]. Therefore, using principles of user-centered design, we developed Cardiometabolic Sutter Health Advanced Reengineered Encounter (CM-SHARE), a web-based application designed to simplify care delivery for patients with cardiometabolic conditions (DM, hypertension, and dyslipidemia) [22]. CM-SHARE extracts essential health data elements from the EHR in real time at the point of care and displays them in novel ways for both physicians and patients. The main features of CM-SHARE include a snapshot view, graphs, medication dispensing history, and risk calculators, where the snapshot view provides an intuitive overview of patient-specific data gathered from different areas that are critical to review for patients with cardiometabolic conditions, whereas graphs and medication dispensing history use graphic views of longitudinal laboratories, vitals, medication dispense and adherence history, and risk calculators that allow physicians to change the values of different risk factors to help educate patients on how changes that modify different risk factors can affect cardiovascular risk. The primary design intent of CM-SHARE is to reduce the time physicians spend “hunting and clicking” for information in the EHR. A previous study showed that CM-SHARE, a voluntary-use digital health solution, was successfully integrated into a real-world primary care setting with high adoption and consistent use in caring for patients with cardiometabolic conditions [12]. In this study, following principles of the digital health technology development and deployment [23,24], we first tested CM-SHARE among a small group of pilot users (ie, PCPs) before spreading to a much broader group of PCP end users. Although there is an increasing availability of digital tools created by health care or high-technology companies, little is known about whether the impact of these digital tools on pilot users is sustained when the technology is spread to a much broader group of users [25,26]. Therefore, we assess CM-SHARE’s impact on physician workflow and whether CM-SHARE achieves its intended goal of improving provider EHR and encounter efficiency in the pilot users and then assess whether similar impacts are sustained after disseminating to a broader group of PCPs.

Clinical workflow and EHR have typically been measured using time-motion studies, which are costly and not scalable [27-30]. In recent years, EHR audit log data have been increasingly used as an alternative approach to estimate the workflow and time used in the EHR, and this approach is scalable and reproducible [31-34]. The audit logs record who (user), when (time), where (location), and what EHR function has been used and are routinely collected in health care systems [33-36]. EHR audit data have been used previously in emergency departments [36] and specialty settings [37,38] to assess the efficiency and have been validated as a resource for analyzing workflows [39,40].

### Objectives

The objective of this study was to thoroughly evaluate the impact of CM-SHARE on physicians’ clinical workflow in primary care encounters. We conducted data collection and analyses in the following order: time-motion observations to develop and validate audit data–derived clinical workflow algorithms, extrapolated for non-EHR clinical work (eg, patient examination and conversation with patients), followed by the application of these algorithms to clinical workflow analysis among CM-SHARE pilot users, and, finally, clinical workflow analysis among a broader group of “spread users” to whom CM-SHARE was made available. Our analysis focused on assessing the impact of CM-SHARE on physician workflows and EHR use. We hypothesized that CM-SHARE’s visual display of health data would (1) reduce the overall encounter time, EHR time, and physician EHR time in the examination room; (2) result in differential reductions in EHR time based on the scheduled visit length and the encounter primary diagnosis; and (3) lead to observable reductions in pilot users that are sustained when CM-SHARE is spread to a broader group of PCPs.

## Methods

### Overview

There were 3 main components to this study. First, we conducted a time-motion study to collect the main workflow time and duration of primary care encounters observed on 3 randomly selected workdays for 9 PCPs. We used these time-motion data to validate the workflow steps and to refine algorithms that capture the workflow based on the EHR audit log data. Second, we evaluated CM-SHARE using a pre-post parallel design, where cases were defined as encounters in which CM-SHARE was launched. Finally, we estimated the impact of CM-SHARE on the audit data–derived workflow outcomes in the matched cohort. The second and third study components were first conducted on the original set of pilot CM-SHARE users involved in the application development. We then repeated these analyses in the spread phase of the study, in which CM-SHARE was implemented and used by a new, broader group of PCPs who were not involved in any aspect of the original design and pilot phase.

### Validation of Audit Data Workflow

We developed algorithms to identify key steps (eg, check-in), tasks performed (eg, EHR use), locations of tasks (eg, examination room), and roles (eg, nurses and physicians) involved in clinical ambulatory workflows using time-stamped audit log files from the EHR and performed a time-motion observation of 101 encounters from 9 PCPs at 3 different primary care clinics. We compared the time-motion–observed times of key workflow points in a patient encounter with workflow measures from the EHR audit log files. The time-motion data-tracking form can be found in Multimedia Appendix 1.

**Figure 1:**
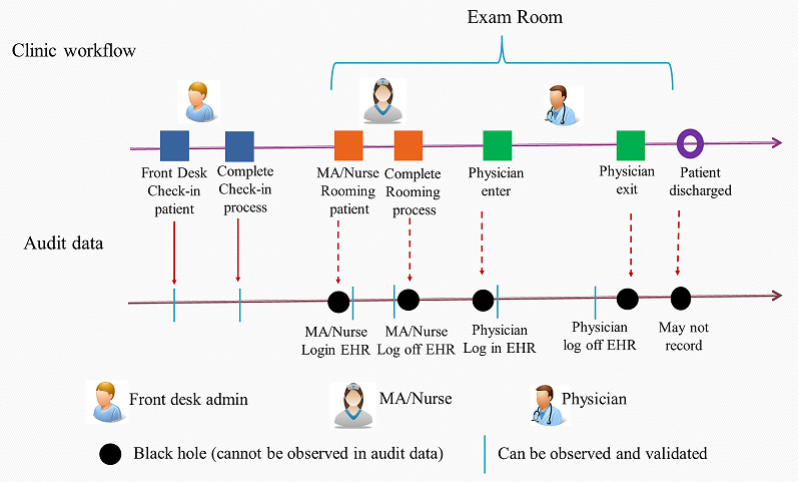
Illustration of outpatient visit workflow and corresponding audit data. EHR: electronic health record; MA: medical assistant.

The critical clinical workflow points included check-in, rooming time (when a medical assistant [MA] or nurse takes a patient to the examination room), nurse exiting the examination room, physician entry into the examination room, physician’s total EHR time in the examination room, physician exiting the examination room, and patient check-out time. We used the term “black holes” to identify essential steps in a clinical workflow, which cannot be observed in the audit data as they do not involve interaction with the EHR but can be observed and recorded in time and motion observations. These black holes include steps such as the physician entering the examination room and the physician exiting the examination room (Figure 1). These are important events to account for as they affect the overall time of the encounter. For example, a physician may enter the examination room and spend several seconds to minutes conversing with the patient before logging into the computer.

Measures that can be reliably observed in audit data include the physician’s start time, accessing the EHR in the examination room, and the time of the last EHR access in the examination room. We compared these key workflow time points recorded in the audit log data with time-motion observations and estimated the distribution of each black hole. We further conducted an ANOVA for each black hole in relation to the EHR user (ie, PCP) and patient. This assessment allowed us to understand the variation among PCPs and among patients. We then imputed the time duration of each black hole based on the distribution, and a random number was generated based on the empirical distribution and imputed for the black hole.

The time duration of each key workflow point was calculated based on the imputed audit data and compared again with the time points from the time-motion observation data. Discrepancies were further analyzed, and audit data for encounters with large discrepancies (discrepancy ≥2 SD) were manually reviewed. EHR activity and time points that were closest to the time recorded in time-motion observations were selected for specific users, and the algorithms used to capture each key workflow time point from the audit data were updated. After this initial validation process, we applied the algorithms to 7474 office encounters on a random working day across all Sutter Health primary care clinics to assess the generalizability. We identified key clinical workflow steps using this method, including check-in time, rooming time, nurse leave time, and physician time in the examination room on the computer. However, in this study, we focused only on the total encounter time (defined as the duration between patient check-in time to the time the patient exits the examination room), the physician’s total time in the examination room, the physician’s time spent in the EHR in the examination room, and the physician’s total EHR click per encounter (Figure 1).

### Study Population

The CM-SHARE application was developed and tested at Sutter Health, a large not-for-profit health system in Northern California that serves a racially and economically diverse patient population. CM-SHARE was implemented in 2 phases. The pilot study was initiated in April 2016 with 6 PCPs from 2 different primary care clinics. These PCPs were involved in the development of the application and had frequent communication (once a month in the first year after the initial launch) with the study team during the pilot-testing period. The spread of CM-SHARE started in October 2019 to a new group of PCPs at a large Sutter medical group that previously did not have access to CM-SHARE. In contrast, these new PCPs were lightly touched by the study team; for example, they were offered group (as opposed to one-on-one training as in the pilot phase) training using a CM-SHARE user manual or training provided by designated EHR trainers who were responsible for training clinicians on all EHR features and capabilities, not just CM-SHARE. Spread users were informed that CM-SHARE was specifically developed for patients with cardiometabolic conditions. When and for whom CM-SHARE was used was completely voluntary and up to a physician’s discretion. Details of CM-SHARE features and adoption have been previously published [22].

At the patient level, primary care patients who had at least one cardiometabolic condition and had at least one visit with pilot PCPs or spread PCPs during the pilot test period or spread period were eligible for the study.

### Outcomes

To assess efficiency, we measured (1) physician’s total time in the examination room, (2) physician’s EHR time in the examination room, (3) total encounter time (ie, from check-in to check-out), and (4) total physician clicks in the EHR for an encounter [41].

As illustrated in Figure 1, the physician’s total time in the examination room was defined as the time between when the physician entered and exited the examination room, estimated based on audit data and imputed data for black holes described previously in the time-motion explanation. The time at which the physician entered the examination room was estimated by identifying the first time the physician logged into the EHR in the examination room, subtracting the imputed value for a black hole (ie, the time between the physician entering the examination room and logging into the EHR), and the physician exit time was estimated based on the EHR log-off time by the physician in the examination room and accounting for the imputed black hole (ie, the time between physician EHR log-off time and the exit examination room time). The physician’s EHR time, estimated based on audit data, was defined as the cumulative time the physician spent in the EHR in the examination room, which is a subset of the physician’s total time spent physically present in the examination room. The total number of physician EHR mouse clicks for each encounter, captured using audit data, was defined based on the cumulative number of EHR log entries (which record the EHR features accessed by a user) for a physician for a given encounter, including all previsit EHR activities (preparation for the visit), during the visit, and postvisit EHR activities (eg, clicking on a diagnosis, on medication tags in EHR, and clicking on a patient message). The total number of clicks reflects EHR information searching and access, and usually, more clicks implied more complex encounters as more patient medical information was reviewed.

### Study Design

#### Overview

**Figure 2:**
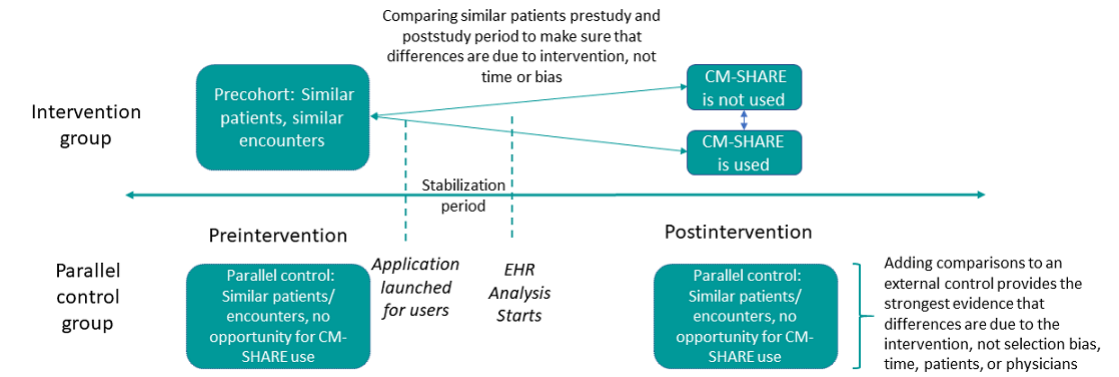
Pre-post matched study design for CM-SHARE evaluation. CM-SHARE: Cardiometabolic Sutter Health Advanced Reengineered Encounter; EHR: electronic health record.

A pre-post matched design was applied, in which we defined the prestudy period as 12 months before the initial CM-SHARE launch in April, 2016, and the CM-SHARE stabilization time as at least 2 months after the initial launch time where the data were not used in the evaluation. The poststudy period was defined as 12 months after the stabilization period (Figure 2). Owing to the disruption of the COVID-19 pandemic and to allow for the stabilization of CM-SHARE use, we identified spread users who consistently used CM-SHARE starting in May 2020 (12 months after the initial spread) as stable CM-SHARE users. Data from October 15, 2018, to October 14, 2019, were used as the prestudy period to determine matched physicians. We compared efficiency measures for encounters occurring between May 2020 and December 2020.

#### Propensity Matching

There are two levels of endogeneity, which we accounted for in part via propensity matching. At the first level, physicians who had used CM-SHARE regularly (ie, used it at least once a week), denoted as case PCPs, may be systematically and unobservably different from those who did not regularly use CM-SHARE. To that end, case PCPs were matched to other Sutter-wide PCPs based on physician panel information, assuming that a physician’s clinical practice pattern, including EHR use and information access, was affected by the composition of their patient panel. A propensity score–matching method was used to identify matched physicians, and a separate method was used to identify matched patients. At the physician level, a logistic regression model was developed, in which covariates included annual patient volume, mean number of appointments per day, average age of the patient (at time of encounter, categorized into <30, 30-49, 50-64, and ≥65 years), proportion of female patients, proportion of different ethnicities (Hispanic and non-Hispanic) in the patient panel, proportion of patients with each cardiometabolic condition (DM, dyslipidemia, and hypertension), practice type (family medicine and internal medicine), and proportion of patients with different levels of morbidity based on the Charlson Comorbidity Index (0, 1-2, and ≥3). The model outcome was the CM-SHARE pilot status (yes or no). Greedy matching was used in this study.

At the second level, patients for whom CM-SHARE was used may also be unobservably different from those for whom CM-SHARE was not used. Therefore, within the pilot physicians and matched control physicians, we performed one-to-one matching at the patient level to determine cases (for which CM-SHARE was used) and control patients (within the matched physicians). The case patients, for whom CM-SHARE was used, were matched to the control patients at the prestudy period and separately at the poststudy period.

Patients were grouped into pre- or poststudy period based on the first visit date in the prestudy period and poststudy period. In the prestudy period, eligible patients included those who had encounters with pilot physicians and matched control physicians. In the poststudy period, control patients were those who were eligible and had encounters with matched control physicians. Data from the prestudy period were used for prestudy period matching, and poststudy period data were used for poststudy period matching.

We developed a separate logistic regression model for patients where the covariates were individual patient-level features, including age, sex, race, ethnicity, percentage of 15-minute scheduled appointments, percentage of 30-minute scheduled appointments, percentage of the level of service (LOS) level 3 visits, percent of LOS level 4 visits, percentage of DM as primary encounter diagnosis, percentage of hypertension as primary encounter diagnosis, and percentage of dyslipidemia as primary encounter diagnosis. The outcome of this model was the CM-SHARE launch status in any patient encounter (yes or no). Greedy matching was also used.

We further defined eligible encounters to require that the encounter diagnoses contain at least one cardiometabolic diagnosis code, and encounters were made by matched case and control patients in the pre- and postperiod. EHR audit data for the eligible encounters were extracted and used in the analysis, and outcome measures were derived from those encounters.

### Stratification

Furthermore, based on the feedback from pilot PCPs on the different use cases for CM-SHARE and guidelines on the content and services provided at ambulatory encounters, the complexity of a patient visit and documentation requirements were usually reflected by LOS or length of the appointment [42]. We expected EHR and CM-SHARE utility to differ according to LOS or length of the appointment. Therefore, we stratified the encounters according to the scheduled length of appointments. To compare the workflow measures, we first estimated the mean difference (and 95% CI) between cases and matched controls in the prestudy period and separately in the poststudy period. We used 2-tailed *t* tests to test the mean difference between prestudy period control versus case patients, poststudy period control versus case patients, and prestudy period controls and poststudy period controls. The 95% CI was estimated by fitting a mixed linear regression model that matched the case and control patients treated as the same cluster. The cluster was taken as the random effect in the model. In addition, the primary diagnosis usually reflects the main reason for the visit; thus, we conducted the abovementioned analysis stratified by the primary diagnosis and assessed whether the impact of CM-SHARE varies according to the reason for the visit.

Sutter Health uses an instance of Epic Systems software (Epic Systems Corporation) as its EHR [24]. All analyses were performed using SAS Enterprise Guide 7.1 (SAS Institute).

### Ethics Approval

This study was reviewed and approved by the Sutter Health Institutional Review Board (IRB #: 833549).

## Results

### Validation Results

Among the 101 encounters, time-motion observations recorded check-in start and end times for all encounters, rooming start time, time the physician enters the examination room, and patient check-out time for >91 (90%) encounters. MA or nurse EHR log-in and log-off times in the examination room and physician EHR log-in and log-off times were collected for 72 to 76 (71%-76%) encounters, respectively. Physician exiting examination room time was collected for 88% (89/101) of the encounters (Multimedia Appendix 2). We were able to capture all EHR workflow time points from the audit log data. Table 1 shows the summary statistics of the differences (time-motion–observed time and time derived from audit log data) for each workflow time point.

**Table 1:** Summary statistics for the difference between time-motion–observed time and time derived from audit log data (N=101).

Workflow event	Encounter, n (%)	Observed and audit data	
		Values, mean (SD)	Values, median (IQR)
Check-in start	101 (100)	0.6 (1.5)	0.3 (0.1 to 0.7)
Check-in end	101 (100)	1.0 (2.9)	0.2 (−0.1 to 1.0)
Room start	—^a^	—	—
MA^b^ logs in	76 (75)	0.2 (1.7)	0.4 (−0.1 to 0.9)
MA logs off	79 (78)	0.1 (0.5)	0.3 (−0.1 to 0.7)
Physician enters	—	—	—
Physician logs in	76 (75)	−0.2 (1.5)	0.2 (−0.6 to 1.0)
Physician logs off	72 (71)	0.3 (1.9)	−0.1 (−0.5 to 0.9)
Physician exits	87 (86)	—	—
Observed time between physician log-off and physician exit time	89 (88)	0.8 (1.3)	0.3 (0.1 to 0.7)

^a^Not available in audit log data; represents a workflow “black hole,” as illustrated in Figure 1.

^b^MA: medical assistant.

Audit data can capture most clinical workflow time points with high accuracy. The difference between time-motion–observed time and time derived from audit data is <1 minute, and for most time points, the difference is <30 seconds. As shown in Table 1, the largest differences between time-motion time and audit data–derived time were for check-in start (mean 0.6, SD 1.5) and end times (mean 1.0, SD 2.9) as time-motion time recorded the time the patient was called to the front desk to check in and the time the patient left the front desk, whereas audit data recorded the time front desk MAs started the EHR check-in process. We also observed a large variation in the patient exiting the examination room time, with a mean of 2.9 (SD 4.1) minutes, as the time patients exit the examination room was not available in the audit data, and we used the last EHR log-off time by the physician in the examination room as the surrogate to compare with time-motion observations.

As illustrated in Figure 1, we calculated the duration for each black hole based on time-motion data. The duration of these “black holes” varies from approximately half a minute to approximately 2.6 minutes, where the black holes associated with MAs are short (<1 minute in most encounters), and the longest unobserved black hole was the interval between the physician entering the examination room and the EHR log-in (mean duration 4.6, SD 5.6 minutes). ANOVA showed that physicians explained 62% of the duration variance, and patient demographic factors explained approximately 15%. The mean duration between physician exit and patient completion of the encounter was 2.7 (SD 4.1) minutes, and the physician explained 39% of the variance, and 17% was explained by patient factors (Table 2).

**Table 2:** Observed duration for each black hole using time-motion data and output from ANOVA.

“Black hole”	Time interval (minute), mean (SD)	Variation explained by clinical staff (%)	Variation explained by patient characteristics^a^ (%)
MA^b^ room to MA log-in	0.73 (1.01)	11.6	35.1
MA log-off to exit	0.51 (1.39)	6.9	46.7
Physician entering examination room to log in	2.62 (1.61)	62.2	14.8
Physician log-off to exit	0.82 (1.29)	11.1	23
Physician exit to patient exit	2.72 (4.11)	39.1	17.5

^a^Patient age, sex, race, and ethnicity.

^b^MA: medical assistant.

### Results Among Pilot Users

We found 6 matched control physicians for only 50% (3/6) of pilot physicians. Among the matched physicians, in the pre–CM-SHARE period, 870 control patients associated with 2845 encounters were matched with the same number of patients (870 encounters where CM-SHARE was launched for their encounters). In the poststudy period, 727 patients associated with 852 encounters during which CM-SHARE was launched (cases) were matched with 727 control patients associated with 3754 encounters.

Among all the eligible encounters with patients with cardiometabolic conditions (N=6599; ie, 2845+3754), <10% (595/6599, 9.02%) of the encounters also had a cardiometabolic condition (DM, hypertension, and dyslipidemia) listed as a primary diagnosis of the encounter.

As shown in Table 3 (additional table in Multimedia Appendix 3), the total encounter time was slightly shorter (mean −2.7, SD 1.1 minutes, 95% CI −4.7 to −0.9; mean −1.6, SD 1.1 minutes, 95% CI −3.2 to −0.1) for cases compared with prestudy period controls, as well as for poststudy period controls for 15-minute appointments only, but not for 30-minute appointments. The time saved may be explained by the reduction in the total EHR time for physicians in the examination room, in which CM-SHARE saves approximately 2 minutes (mean −2.0, SD 1.3 minutes; 95% CI −3.4 to −0.9) compared with controls in the prestudy period and a similar amount of time in the poststudy period (mean −1.9, SD 0.9 minutes; 95% CI −3.8 to −0.5). CM-SHARE had no impact on physicians’ total time in the examination room or on physicians’ total EHR clicks.

**Table 3:** Summary of difference in time and 95% CI in comparing controls versus cases workflow measures during pilot period.

Workflow measure and scheduled appointment time (minutes)		Difference between matched control in preperiod and matched cases (case and control; n=788)				Difference between matched control in postperiod and matched cases (case and control; n=669)		
		Values, n (%)	Mean (95% CI)	*P* value	Values, n (%)		Mean (95% CI)	*P* value
**Total encounter time (minutes)**								
	≤20	325 (41.2)	−2.7 (−4.7 to −0.9)	.002	310 (46.3)		−1.6 (−3.2 to −0.1)	.02
	≥30	463 (58.8)	−0.6 (−3.1 to 2.2)	.11	359 (53.7)		−0.3 (−2.4 to 1.1)	.18
**Total physician time in the examination room (minutes)**								
	≤20	325 (41.2)	−0.6 (−1.9 to 2.0)	.35	310 (46.3)		0.5 (−0.7 to 3.2)	.46
	≥30	463 (58.8)	−1.0 (−2.9 to 2.4)	.41	359 (53.7)		−0.7 (−2.1 to 2.0)	.29
**Physician EHR^a^ time in the examination room (minutes)**								
	≤20	325 (41.2)	−2.0 (−3.9 to −0.9)	.006	310 (46.3)		−1.9 (−3.8 to −0.5)	.009
	≥30	463 (58.8)	−1.3 (−3.4 to 0.5)	.12	359 (53.7)		−1.1 (−3.1 to 0.7)	.15
**Physician total clicks in the EHR**								
	≤20	N/A^b^	N/A	N/A	N/A		6 (−24 to 7)	.29
	≥30	N/A	N/A	N/A	N/A		12 (−27 to 10)	.33

^a^EHR: electronic health record.

^b^N/A: not applicable.

However, there was a significant reduction in the total encounter time, total physician time in the EHR, and total physician EHR clicks for two subsets of encounters: encounters with DM as the primary diagnosis and encounters with hypertension as the primary diagnosis. For diabetes encounters, the average total encounter time was 51.3 (SD 5.7) minutes and 49.5 (SD 5.4) minutes for controls in the prestudy period and the poststudy period, respectively, and was reduced to 47.6 (SD 5.1) minutes in CM-SHARE encounters (Multimedia Appendix 4). The mean reduction was 2.1 to 3.5 minutes within matched pairs (Table 4). A substantial reduction was observed in total physician EHR time for diabetes and hypertension encounters in the CM-SHARE group, showing an approximately 30% reduction (the reduction was approximately 2.9-3.5 minutes for hypertension and 4.1-4.3 minutes for diabetes encounters; Table 4). Physician clicks within the EHR were also reduced significantly when using CM-SHARE by 25% in hypertension encounters (from 173 to 129) and 14% for diabetes encounters (from 126 to 108; Multimedia Appendix 4; Table 4).

**Table 4:** Summary of difference in time and 95% CI in comparing controls versus cases workflow measures during the pilot period.

Workflow measure and primary diagnosis			Difference between matched control in prestudy period and matched cases (case and control) (n=283)							Difference between matched control in poststudy period and matched cases (case and control) (n=312)				
			Value, n (%)		Mean (95% CI)		*P* value		Value, n (%)			Mean (95% CI)		*P* value
**Total encounter time (minutes)**														
	Diabetes	129 (45.6)		−2.8 (−3.9 to −0.2)		.03		145 (46.5)			−2.1 (−3.7 to −0.1)		.03	
	Hypertension	114 (40.3)		−3.5 (−5.2 to −0.1)		.04		124 (39.7)			−3.4 (−4.9 to −0.9)		.008	
	Hyperlipidemia	40 (14.1)		−3.7 (−6.2 to 1.8)		.19		43 (13.8)			−4.2 (−5.9 to 0.6)		.09	
**Total physician time in the examination room (minutes)**														
	Diabetes	129 (45.6)		−2.1 (−3.9 to 0.8)		.12		145 (46.5)			−2.2 (−3.7 to 0.7)		.15	
	Hypertension	114 (40.3)		−0.5 (−1.2 to 1.3)		.36		124 (39.7)			−0.6 (−1.3 to 1.2)		.57	
	Hyperlipidemia	40 (14.1)		−0.1 (−1.0 to 1.7)		.61		43 (13.8)			1.0 (−0.7 to 2.3)		.15	
**Physician EHR^a^ time in the examination room (minutes)**														
	Diabetes	129 (45.6)		−4.3 (−5.2 to −2.4)		.007		145 (46.5)			−4.1 (−5.0 to −2.6)		.005	
	Hypertension	114 (40.3)		−2.9 (−3.8 to −0.5)		.02		124 (39.7)			−3.5 (−4.6 to −1.4)		.009	
	Hyperlipidemia	40 (14.1)		−2.2 (−3.7 to 1.0)		.14		43 (13.8)			−1.4 (−3.1 to 0.8)		.10	
**Physician total clicks in EHR**														
	Diabetes	N/A^b^		N/A		N/A		145 (46.5)			−19 (−34 to −2)		.04	
	Hypertension	N/A		N/A		N/A		124 (39.7)			−50 (−70 to −22)		.006	
	Hyperlipidemia	N/A		N/A		N/A		43 (13.8)			4 (−11 to 15)		.35	

^a^EHR: electronic health record.

^b^N/A: not applicable.

### Results Among Spread Users

We matched 48 CM-SHARE spread physicians with 84 control physicians and further matched 1272 patients in the CM-SHARE group with 3412 control patients, associated with 1119 and 4240 eligible encounters, respectively. As shown in Multimedia Appendix 5 and Table 5, a significant reduction in total encounter time for the CM-SHARE group was only observed for encounters with appointments ≤20 minutes (5.3-minute reduction on average) but not for encounters with longer appointment times. However, the total physician’s EHR time was significantly reduced for both longer and shorter appointments (reduced by 17%-31%, respectively), and a 16% reduction was observed in physicians’ total clicks for both longer and shorter appointments.

Furthermore, <10% of eligible encounters had cardiometabolic as the primary diagnosis. Owing to the limited sample size for matched cases, we only observed a reduction in total encounter time for diabetes as the primary diagnosis (mean −3.2, 95% CI −4.9 to −0.9) and, to a lesser degree, for hypertension encounters (mean −2.9, 95% CI −4.0 to −0.1). For hypertension encounters, we also observed an approximately 33% reduction of physician EHR time in the examination room (mean −2.1, 95% CI −4.7 to −0.2) and a 19% reduction in physician total EHR clicks (mean −24, 95% CI −38 to −12; Multimedia Appendix 6; Table 6).

**Table 5:** Summary of differences in time and 95% CI in comparing controls versus cases for the encounter-related workflow measures in the Cardiometabolic Sutter Health Advanced Reengineered Encounter spread period, stratified by scheduled appointment time.

Workflow measure and scheduled appointment time (minutes)		Difference between matched control in postperiod and matched cases (case and control)	
		Values, mean (95% CI)	*P* value
**Total encounter time (minutes)**			
	≤20	−5.3 (−7.5 to −0.7)	.002
	≥30	3.7 (−6.1 to 3.9)	.37
**Total physician time in the examination room (minutes)**			
	≤2	−2.0 (−4.2 to 1.7)	.15
	≥30	3.4 (−4.9 to 2.1)	.23
**Physician EHR^a^ time in the examination room (minutes)**			
	≤20	−4.0 (−5.7 to −1.8)	<.001
	≥30	−2.1 (−4.5 to −0.3)	.003
**Physician total clicks in EHR**			
	≤20	−11 (−17 to −2)	.02
	≥30	−13 (−19 to −4)	.008

^a^EHR: electronic health record.

**Table 6:** Summary of difference in time and 95% CI in comparing controls versus cases workflow measures during spread period, stratified by primary diagnosis.

Workflow measure and primary diagnosis			Difference between matched control in postperiod and matched cases (case and control)						
			Values, n (%)				Mean (95% CI)		*P* value
			Case (n=132)		Control (n=353)				
**Total encounter time (minutes)**									
	Diabetes	54 (40.9)		157 (44.5)		−3.2 (−4.9 to −0.9)		.01	
	Hypertension	41 (31.1)		144 (40.8)		−2.9 (−4.0 to −0.1)		.04	
	Hyperlipidemia	37 (28.0)		52 (14.7)		−3.9 (−6.7 to 1.1)		.14	
**Total physician time in the examination room (minutes)**									
	Diabetes	54 (40.9)		157 (44.5)		−1.5 (−4.2 to 1.9)		.51	
	Hypertension	41 (31.1)		144 (40.8)		−1.9 (−5.2 to 3.7)		.49	
	Hyperlipidemia	37 (28.0)		52 (14.7)		−1.4 (−5.0 to 3.4)		.78	
**Physician EHR^a^ time in the examination room (minutes)**									
	Diabetes	54 (40.9)		157 (44.5)		−3.3 (−7.0 to 0.3)		.12	
	Hypertension	41 (31.1)		144 (40.8)		−2.1 (−4.7 to −0.2)		.03	
	Hyperlipidemia	37 (28.0)		52 (14.7)		−0.3 (−4.2 to 3.5)		.89	
**Physician total clicks in EHR**									
	Diabetes	54 (40.9)		157 (44.5)		−13 (−24 to −3)		.02	
	Hypertension	41 (31.1)		144 (40.8)		−24 (−38 to −12)		.009	
	Hyperlipidemia	37 (28.0)		52 (14.7)		−7 (−24 to 10)		.51	

^a^EHR: electronic health record.

## Discussion

### Principal Findings

We successfully used EHR audit data to evaluate efficiency (time and clicks) in the EHR for physicians using a new web-based application and showed that physician EHR time in the examination room was reduced by 17% to 31%, and clicks in the EHR were reduced by 14% to 25%, varying by characteristics of the encounter (ie, scheduled length of the appointment and primary diagnosis of the encounter) when using the web-based application in a pilot phase. More importantly, we also replicated our method for evaluating efficiency with a group of spread users and found similar reductions in time and clicks on the computer in the examination room for both long and short appointments. Few studies report testing on validity [32] similar to ours, and when compared with them, the workflow times derived from audit data in our study are consistent with theirs [40,43,44]. Our study further offers a methodology for using audit data to evaluate the impact of a clinical decision support tool on physician workflow and efficiency.

### Comparison With Prior Work

CM-SHARE development was motivated by the desire to design a dashboard with better data integration and an intuitive visual display to facilitate physician decisions and communication with patients [22]. We previously assessed the adoption in the pilot phase and identified the target patient population for which the CM-SHARE is most likely to be used [22]. In this study, the reduction in total EHR time using CM-SHARE was seen not only during pilot testing with physicians but also in the spread physicians who were neither individually trained as in the pilot phase nor frequently interacted with by the CM-SHARE study team. This demonstrates that the initial design intention of CM-SHARE was fulfilled. Furthermore, we observed a significant reduction in physician EHR time in the examination room and no change in the total physician time in the examination room, implying that physicians are likely to have more time to communicate with patients, improving patient satisfaction. From qualitative data taken during the initial pilot [22], we know that 2 pilot providers describe using CM-SHARE for patient education and that users describe CM-SHARE as leading to better discussions with patients, which seems to hold true in spread physicians and may explain the reduction in time spent on the computer with no changes in overall time spent with the patient.

These findings in the initial pilot users and spread sites further show that CM-SHARE is more valuable for patients with diabetes or hypertension-related encounters and less valuable for patients with dyslipidemia. Hyperlipidemia is a common comorbidity for hypertension and diabetes, and the management of dyslipidemia usually occurs either when patients encounter hypertension or diabetes or as one component in encounters related to cardiovascular disease management or prevention [45,46]. Standalone hyperlipidemia encounters are less common than those of hypertension or diabetes; therefore, it is less likely to detect a time reduction in hyperlipidemia-related encounters. In contrast, compared with diabetes-related encounters, physician EHR time for hypertension-related encounters is much shorter (10 minutes for hypertension vs 14 minutes for diabetes in controls), implying that less EHR information is required for physicians to manage patients with hypertension compared with patients with diabetes. We observed a similar percentage (4/14, 4/11, or ~30%) of physician EHR time saved by using CM-SHARE for those 2 conditions, indicating that the information that affects clinical decisions, EHR processes, and clinical tasks for an encounter may not vary significantly for those 2 cardiometabolic conditions. It also suggests considering target population needs when designing and implementing clinical support tools to optimize the benefits to those populations.

Interestingly, among pilot users, we only observed CM-SHARE reducing physicians’ EHR time during encounters with shorter scheduled appointment lengths (ie, ≤20 minutes) and not for longer appointments (ie, ≥30 minutes); however, a reduction in EHR time for both long and short appointments was observed in spread physicians. On the basis of the American Academy of Family Physicians and Centers for Medicare and Medicaid Services guidelines on the evaluation and management of office visits [42], for appointment lengths <20 minutes, the number and complexity of problems needing to be addressed during the encounter and the complexity of data to be reviewed were relatively fewer or simpler, whereas ≥ 30-minute appointments usually imply more problems to be addressed and, thus, more patient data to be reviewed. Longer appointments are usually used for more complex patients with more comorbidities [47]. Given the EHR content provided by CM-SHARE, which mostly includes the information directly related to cardiometabolic conditions, and, to a lesser degree, on comorbidities, we expect that CM-SHARE’s features may not be sufficient to produce similar reductions in outcomes when addressing comorbidities other than cardiometabolic conditions. It is surprising that CM-SHARE reduces EHR time for longer appointments in spread physicians but not in pilot physicians, in addition to the larger sample size for the spreading evaluation. A possible reason is that the practice pattern and use of CM-SHARE may differ between pilot physicians and spread physicians. Pilot physicians participated in the tool design and were well aware of the target condition that CM-SHARE was designed for, the limitations of CM-SHARE, and the “design thinking” that may drive them more likely to go back to the EHR to manage other comorbidities [48], whereas spread physicians may be likely to “think out-of-box” to optimize the utility of CM-SHARE functions and to use the CM-SHARE as a tool to manage common risk factors for patients with comorbidities. More studies, especially qualitative interviews, are needed to understand this discrepancy between physicians and the difference in whether and how CM-SHARE is used in short and long appointments.

In summary, CM-SHARE has shown improvement in data integration and reduction of EHR time for certain encounters (eg, encounters with a shorter scheduled appointment time and encounters with chief complaints of diabetes and hypertension). A similar design and evaluation approach (eg, user-centered design, workflow integration, and pseudoexperimental design) has the potential to be generalized to other similar clinical decision support tools deployed in real-world settings. If similar improvements in the physician EHR efficiency are observed in other studies, it will provide great insight into redesigning the EHR user interface and reorganizing disease-related contents with a better, more user-friendly visual display.

### Limitations

This study had several limitations. First, this study was conducted at a single center with a single EHR system, and the results may not be directly generalizable to other health care systems or systems with a different EHR vendor [41]. However, the methodology, including the use of audit data, validation, imputation, and the study design, is generalizable. Second, audit data may overestimate physicians’ EHR time in the examination room. For example, patient examination time or time away from the EHR talking to patients between EHR activities might be counted as EHR time. Finally, this was not a randomized trial, and 2-level matching was applied; therefore, we may not be able to control all confounding variables and nested correlations between physicians and patients, which may be related to EHR efficiency.

### Conclusions

Combining audit log files and clinical information from the EHR, we were able to evaluate the impact of a clinical decision support tool (CM-SHARE) on the clinical workflow times and physicians’ EHR efficiency. The CM-SHARE web-based application significantly reduced physicians’ EHR time in the examination room, particularly for hypertension- or diabetes-related encounters, and less for complex encounters. Our approach offers an innovative way of evaluating digital tools used in clinical settings.

## Appendix

Multimedia Appendix 1 Time-motion data tracking form.

Multimedia Appendix 2 Summary of the number of encounters in time-motion observation and the encounters where audit log data were used in validation.

Multimedia Appendix 3 Summary of time duration for key encounter-related workflow measures and comparison between prestudy and poststudy period for matched cases and controls.

Multimedia Appendix 4 Summary of time duration for key encounter-related workflow measures by primary diagnosis and comparison between prestudy and poststudy period for matched cases and controls during the pilot period.

Multimedia Appendix 5 Summary of time duration for key encounter-related workflow measures and comparison between poststudy period for matched cases and controls in the Cardiometabolic Sutter Health Advanced Reengineered Encounter spread period.

Multimedia Appendix 6 Summary of time duration for key encounter-related workflow measures and comparison between poststudy period for matched cases and controls in the Cardiometabolic Sutter Health Advanced Reengineered Encounter spread period for encounters with Diabetes, Hypertension or Hyperlipidemia.

## Abbreviations

CM-SHARE: Cardiometabolic Sutter Health Advanced Reengineered Encounter
DM: diabetes mellitus
EHR: electronic health record
LOS: level of service
MA: medical assistant
PCP: primary care physician
