# Supplementary material for: Evaluating the Impact of a Point-of-Care Cardiometabolic Clinical Decision Support Tool on Clinical Efficiency Using Electronic Health Record Audit Log Data: Algorithm Development and Validation
Source: JMIR Med Inform. 2022 Sep 6;10(9):e38385. doi: 10.2196/38385 (PMC9490545; doi:10.2196/38385)
Supplement: Multimedia Appendix 1 [file medinform_v10i9e38385_app1.docx]

| **EPIC AUDIT FILE VALIDATION FORM** | | | |
| --- | --- | --- | --- |
| Location: |  | Date:  Appt Time: |  |
| Patient Name: |  | CSN: |  |

**Multimedia Appendix 1.** Time-motion data tracking form.

| **SEQUENCE ORDER** | **USER ID** | **EVENT** | **START TIME** | **DURATION (LAP TIME)** |
| --- | --- | --- | --- | --- |
| 1 |  | Start patient arrival (when they walk through the door) |  | 0:00 |
| 2 |  | PSR^a^ log on |  |  |
| 3 |  | PSRa log off |  |  |
| 4 |  | Collect CSN^b^ |  |  |
| 5 |  | Patient sits down in waiting room |  |  |
| 6 |  | Patient gets called to room |  |  |
| 7 |  | Patient enters room |  |  |
| 8 |  | MA^c^ logs into Epic |  |  |
| 9 |  | MA^c^ logs off Epic |  |  |
| 10 |  | MA^c^ leaves room |  |  |
| 11 |  | Provider enters room |  |  |
| 12 |  | Provider logs into Epic |  |  |
| 13 |  | Provider logs off of Epic |  |  |
| 14 |  | Provider leaves room |  |  |
| 15 |  | Anyone else enters |  |  |
| 16 |  | Anyone else logs into Epic |  |  |
| 17 |  | Anyone else logs off of Epic |  |  |
| 18 |  | Anyone else leaves the room |  |  |
| 19 |  | Patient leaves room |  |  |
| 20 |  | Patient arrives at Front Desk or Lab |  |  |
| 21 |  | Patient leaves clinics |  |  |
| **Total Time** | | | |  |

^a^PSR: patient service representative

^b^CSN: contact serial number

^c^MA: medical assistant
