# Supplementary material for: Evaluating the Impact of a Point-of-Care Cardiometabolic Clinical Decision Support Tool on Clinical Efficiency Using Electronic Health Record Audit Log Data: Algorithm Development and Validation
Source: JMIR Med Inform. 2022 Sep 6;10(9):e38385. doi: 10.2196/38385 (PMC9490545; doi:10.2196/38385)
Supplement: Multimedia Appendix 2 [file medinform_v10i9e38385_app2.docx]

**Multimedia Appendix 2.** Summary of the number of encounters in time-motion observation and the encounters where audit log data were used in validation.

| Workflow event | Time-motion observed (N = 101) | Derived from audit data (N=101) |
| --- | --- | --- |
| Check-in start | 91 (90.1%) | 101 |
| Check-in end | 84 (83.2%) | 101 |
| Room start | 92 (91.1%) | Not directly |
| MA^a^ login | 76 (75.3%) | 101 |
| MA^a^ logoff | 79 (75.3%) | Not measure |
| MA^a^ exit exam room |  |  |
| Physician enter | 96 (95.1%) | Not directly |
| Physician login | 76 (75.3%) | 101 |
| Physician logoff | 72 (71.3%) | 101 |
| Physician exit | 89 (88.1%) | Not directly |
| Patient exit | 91 (90.1%) | Not directly |

^a^MA: medical assistant
