# Supplementary material for: Evaluating the Impact of a Point-of-Care Cardiometabolic Clinical Decision Support Tool on Clinical Efficiency Using Electronic Health Record Audit Log Data: Algorithm Development and Validation
Source: JMIR Med Inform. 2022 Sep 6;10(9):e38385. doi: 10.2196/38385 (PMC9490545; doi:10.2196/38385)
Supplement: Multimedia Appendix 3 [file medinform_v10i9e38385_app3.docx]

**Multimedia Appendix 3.** Summary of time duration for key encounter-related workflow measures and comparison between prestudy and poststudy period for matched cases and controls.

| Workflow Measure | Scheduled Appoint Time (min) | Encounters for Matched control in prestudy period  (N=2,287) | Encounters for Matched control in poststudy period  (N=3,755) | Encounters for Matched Cases in poststudy period  (N=852) | p-value for Pairwise comparison* |
| --- | --- | --- | --- | --- | --- |
| Total Encounter Time (min) | <=20 minutes | (N=1357)  50.1 (5.1) | N=2231  49.1 (5.5) | N=552  47.8 (4.7) | *P1*=.04  *P2*=.05  *P3*=.23 |
|  | >=30 minutes | (N=920)  54.5 (6.4) | N=1542  54.0 (6.1) | N=300  54.0 (6.5) | *P1*=.17  *P2*=.49  *P3*=.11 |
| Total clinician time in the exam room (min) | <=20 minutes | (N=1357)  15.3 (1.4) | N=2231  14.6 (1.9) | N=552  15.0 (1.4) | *P1*=.21  *P2*=.11  *P3*=.09 |
|  | >=30 minutes | (N=920)  19.5 (2.1) | N=1542  19.2 (2.2) | N=300  18.6 (2.5) | *P1*=.09  *P2*=.08  *P3*=.16 |
| Clinician EHR time in exam room (min) | <=20 minutes | (N=1357)  10.4 (2.9) | N=2231  10.1 (2.7) | N=552  8.7 (2.1) | *P1*=.01  *P2*=.02  *P3*=.13 |
|  | >=30 minutes | (N=920)  14.6 (3.2) | N=1542  14.5 (3.1) | N=300  13.8 (2.9) | *P1*=.10  *P2*=.10  *P3*=.37 |
| Total EHR clicks by clinicians | <=20 minutes | - | 108 (32) | 114 (34) | *P2*=.23 |
|  | >=30 minutes | - | 136 (46) | 150 (49) | *P2*= .11 |

**P1*: p-value comparing prestudy period control vs. cases); *P2*: p-value comparing poststudy period control vs. cases; *P3*: p-value comparing prestudy period control and poststudy period control
