# Supplementary material for: Evaluating the Impact of a Point-of-Care Cardiometabolic Clinical Decision Support Tool on Clinical Efficiency Using Electronic Health Record Audit Log Data: Algorithm Development and Validation
Source: JMIR Med Inform. 2022 Sep 6;10(9):e38385. doi: 10.2196/38385 (PMC9490545; doi:10.2196/38385)
Supplement: Multimedia Appendix 4 [file medinform_v10i9e38385_app4.docx]

**Multimedia Appendix 4.** Summary of time duration for key encounter-related workflow measures by primary diagnosis and comparison between prestudy and poststudy period for matched cases and controls during the pilot period.

| Workflow Measure | Scheduled Appoint Time (min) | Encounters for Matched control in prestudy period | Encounters for Matched control in poststudy period | Encounters for Matched Cases in poststudy period | p-value for Pairwise comparison^1^ |
| --- | --- | --- | --- | --- | --- |
| Total Encounter Time (min) | Diabetes | N=135  51.3 (5.7) | N=382  49.5 (5.4) | N=150  47.6 (5.1) | *P1*=.04*  *P2*=.05  *P3*=.10 |
|  | Hypertension | N=246  52.1 (4.4) | N=415  51.9 (4.5) | N=129  48.3 (4.3) | *P1*=.06  *P2*=.08  P3=.29 |
|  | Hyperlipidemia | N=104  53.1 (5.2) | N=145  54.3 (6.1) | N=46  45.5 (5.9) | *P1*=.04*  *P2*=.03*  *P3*=.14 |
| Total clinician time in the exam room (min) | Diabetes | N=135  17.2 (2.5) | N=382  17.4 (2.6) | N=150  15.5 (1.7) | *P1*=.06  *P2*=.05  *P3*=.14 |
|  | Hypertension | N=246  15.0 (1.9) | N=415  14.8 (1.7) | N=129  14.3 (1.5) | *P1*=.10  *P2*=.13  *P3*=.28 |
|  | Hyperlipidemia | N=104  15.5 (2.7) | N=145  14.3 (2.9) | N=46  15.3 (2.9) | *P1*=.33  *P2*=.20  *P3*=.19 |
| Clinician EHR time in exam room (min) | Diabetes | N=135  14.9 (2.1) | N=382  14.5 (2.0) | N=150  10.1 (1.6) | *P1*=.01*  *P2*=.01*  *P3*=.23 |
|  | Hypertension | N=246  10.8 (1.9) | N=415  10.9 (1.8) | N=129  6.8 (2.1) | *P1=.006**  *P2=.004**  *P3*=.32 |
|  | Hyperlipidemia | N=104  13.7 (2.4) | N=145  12.7 (2.6) | N=46  11.1 (2.9) | *P1*=.06  *P2*=.09  *P3*=.15 |
| Clinician total clicks in EHR | Diabetes | - | 126 (29) | 108 (23) | *P2*=.04* |
|  | HTN | - | 173 (37) | 129 (35) | *P2*=.01* |
|  | Hyperlipidemia | - | 134 (31) | 136 (32) | *P2*=.87 |
| ^1^ *P1*: P-value comparing prestudy period control vs. cases; *P2*: P-value comparing poststudy period control vs. cases; *P3*: P-value comparing prestudy period control and poststudy period control  *statistical significance | | | | | |
