# Supplementary material for: Evaluating the Impact of a Point-of-Care Cardiometabolic Clinical Decision Support Tool on Clinical Efficiency Using Electronic Health Record Audit Log Data: Algorithm Development and Validation
Source: JMIR Med Inform. 2022 Sep 6;10(9):e38385. doi: 10.2196/38385 (PMC9490545; doi:10.2196/38385)
Supplement: Multimedia Appendix 5 [file medinform_v10i9e38385_app5.docx]

**Multimedia Appendix 5.** Summary of time duration for key encounter-related workflow measures and comparison between poststudy period for matched cases and controls in the Cardiometabolic Sutter Health Advanced Reengineered Encounter spread period.

| Workflow Measure | Scheduled Appoint Time (min) | Encounters for Matched control in poststudy period  (N=4240) | Encounters for Matched Cases in poststudy period  (N=1119) | P-value for comparison* |
| --- | --- | --- | --- | --- |
| Total Encounter Time (min) | <=20 minutes | N=3355  59.1 (8.7) | N=853  53.4 (7.6) | *P*=.05 |
|  | >=30 minutes | N=885  57.9 (10.3) | N=266  63.7 (15.4) | *P*=.22 |
| Total clinician time in the exam room (min) | <=20 minutes | N=3355  15.8 (8.2) | N=853  13.0 (5.9) | *P*=.19 |
|  | >=30 minutes | N=885  16.0 (10.7) | N=266  20.9 (10.2) | *P*=.11 |
| Clinician Total time in EHR (min) | <=20 minutes | N=3355  12.9 (7.8) | N=853  7.5 (3.7) | *P*=.03 |
|  | >=30 minutes | N=885  12.0 (6.2) | N=266  9.7 (3.1) | *P*=.04 |
| Clinician Total clicks in EHR | <=20 minutes | N=3355  66 (13) | N=853  55 (14) | *P*=.02 |
|  | >=30 minutes | N=885  79 (19) | N=266  66 (20) | *P*=.01 |
