# Supplementary material for: Evaluating the Impact of a Point-of-Care Cardiometabolic Clinical Decision Support Tool on Clinical Efficiency Using Electronic Health Record Audit Log Data: Algorithm Development and Validation
Source: JMIR Med Inform. 2022 Sep 6;10(9):e38385. doi: 10.2196/38385 (PMC9490545; doi:10.2196/38385)
Supplement: Multimedia Appendix 6 [file medinform_v10i9e38385_app6.docx]

**Multimedia Appendix 6.** Summary of time duration for key encounter-related workflow measures and comparison between poststudy period for matched cases and controls in the Cardiometabolic Sutter Health Advanced Reengineered Encounter spread period for encounters with Diabetes, Hypertension or Hyperlipidemia.

| Workflow Measure | Primary Diagnosis | Encounters for Matched control in post-period  (N=353) | Encounters for Matched Cases in post-period  (N=132) | *P*-value for comparison |
| --- | --- | --- | --- | --- |
| **Total Encounter Time (min)** | Diabetes | N=157  52.4 (8.1) | N=54  49.2 (8.9) | 0.01 |
|  | Hypertension | N=144  53.5 (16.2) | N=41  50.5 (17.9) | 0.04 |
|  | Hyperliplidemia | N=52  48.1 (9.1) | N=37  44.1 (7.9) | 0.15 |
| **Total physician time in the exam room (min)** | Diabetes | N=157  15.7 (2.3) | N=54  14.2 (2.1) | 0.53 |
|  | Hypertension | N=144  16.9 (3.2) | N=41  15.0 (3.4) | 0.47 |
|  | Hyperliplidemia | N=52  16.0 (2.8) | N=37  14.7 (3.0) | 0.78 |
| **Physician Total time in EHR (min)** | Diabetes | N=157  12.1 (3.8) | N=54  8.7 (3.4) | 0.09 |
|  | Hypertension | N=144  9.5 (3.6) | N=41  6.1 (3.0) | 0.02 |
|  | Hyperliplidemia | N=52  8.3 (3.9) | N=37  7.9 (3.4) | 0.81 |
| **Physician Total clicks in EHR** | Diabetes | N=157  110 (19) | N=54  97 (17) | 0.02 |
|  | Hypertension | N=144  129 (27) | N=41  105 (21) | 0.01 |
|  | Hyperliplidemia | N=52  129 (28) | N=37  121 (30) | 0.51 |
